# Study on a New Electromagnetic Flow Measurement Technology Based on Differential Correlation Detection

**DOI:** 10.3390/s20092489

**Published:** 2020-04-28

**Authors:** Liang Ge, Junxian Chen, Guiyun Tian, Wen Zeng, Qi Huang, Ze Hu

**Affiliations:** 1 College of Mechanical and Electronic Engineering, Southwest Petroleum University, Chengdu 610500, China; cgroad@swpu.edu.cn (L.G.); 201822000307@stu.swpu.edu.cn (J.C.); 201731034414@stu.swpu.edu.cn (Q.H.); huze@swpu.edu.cn (Z.H.); 2 Institute for Artificial Intelligence, Southwest Petroleum University, Chengdu 610500, China; 3 School of Engineering, Newcastle University, NE1 7RU Newcastle, UK; 4 College of materials science and Engineering, Chongqing University, Chongqing 400044, China; wenzeng@cqu.edu.cn

**Keywords:** electromagnetic flowmeter, correlation detection, differential amplification, weak signal detection

## Abstract

Under the conditions of low flow rate and strong noise, the current electromagnetic flowmeter (EMF) cannot satisfy the requirement for measurement or separate the actual flow signal and interference signal accurately. Correlation detection technology can reduce the bandwidth and suppress noise effectively using the periodic transmission of signal and noise randomness. As for the problem that the current anti-interference technology cannot suppress noise effectively, the noise and interference of the electromagnetic flowmeter were analyzed in this paper, and a design of the electromagnetic flowmeter based on differential correlation detection was proposed. Then, in order to verify the feasibility of the electromagnetic flow measurement system based on differential correlation, an experimental platform for the comparison between standard flow and measured flow was established and a verification experiment was carried out under special conditions and with flow calibration measurements. Finally, the data obtained in the experiment were analyzed. The research result showed that an electromagnetic flowmeter based on differential correlation detection satisfies the need for measurement completely. The lower limit of the flow rate of the electromagnetic flowmeter based on the differential correlation principle could reach 0.084 m/s. Under strong external interferences, the electromagnetic flowmeter based on differential correlation had a fluctuation range in output value of only 10 mV. This shows that the electromagnetic flowmeter based on the differential correlation principle has unique advantages in measurements taken under the conditions of strong noise, slurry flow, and low flow rate.

## 1. Introduction

The electromagnetic flowmeter (EMF) is a measurement apparatus based on Faraday’s law for measuring the volume flow of conductive liquids. It is widely used in the petroleum and chemistry industries as well as in food and medicine manufacturing, and also in sewage processing, channel flow measurement, and reservoir resource transfer [1,2,3]. The electromagnetic flowmeter has some advantages over other traditional flow measurement meters, such as having no movable parts that obstruct the flow; no pressure loss; and showing little influence of temperature, density, and pressure on the measurement of the conductive flow [4]. Under special conditions, the amplitude of the induction electromotive signal of the EMF is only a few millivolts or even smaller. Meanwhile, the difficulty in extracting an effective signal increases because of various interferences in the electrodes. Since the traditional EMF cannot distinguish between the actual flow signal and interference noise, thereby only eliminating one type of interference, interference noise cannot be removed synchronously. Inappropriate noise elimination even introduces new interference to the system, leading to a lower measurement accuracy [5,6,7]. Therefore, great significance is attached to improving the signal-to-noise ratio, distinguishing interference components, and eliminating interference. 

The inter-electrode interference of the EMF mainly includes differential interference, in-phase interference, power-line interference, electro-chemistry interference, and slurry fluid interference. The improvement of the EMF measurement accuracy also optimizes anti-interference technology [8,9,10]. Liang proposed a function model of amplitude probability density to study the flow signal, in order to reveal the influence of the flow signal and noise in describing the characteristics of the flow signal. However, this method only worked on eliminating slurry interference and the numerical fitting was very complex [11]. Linnert, M. proposed a Kalman filtering method based on the state space model of the sensor, which separated the current proportional signal from the electrode voltage drift with noise. However, this method was only aimed at voltage drift caused by the electrochemical reaction. A long period of time was needed in this method and new errors could be introduced to the system [12]. Li, Y.W. established a prediction model between different excitation structure parameters and performance evaluation indexes based on the neural network to improve the accuracy of the electromagnetic flowmeter. However, the stable part of the signal had to be maintained for a sufficient period of time to extract effective data by excitation, which easily caused a large power consumption of the EMF and was not conducive to eliminating the slurry interference [13]. Webilor, R.O. introduced a new dual-frequency induction flow tomography system based on the multi-electrode electromagnetic flowmeter. However, the electrode structure was very complex, the speed field needed continuous optimization, and a high-quality double-frequency excitation system was needed, which definitely lowered the zero-point stability [14]. Lehtikangas, O. proposed a method to optimize the coil current and produce a magnetic field, and estimated the speed field accurately. However, optimum excitation was needed for the coil optimization, which took a long time. This method was only suitable for an axial symmetry flow experiment [15]. Wu, J.P. proposed a method whereby the transient process of the EMF was studied to measure the flow rate so as to create an application requiring ultra-low power consumption. A large amount of offline data analysis was needed in this method, and the elimination of differential interference was mainly studied [16].

According to investigation and analysis, although some progress has been made in the anti-interference technology of the EMF, synchronous noise reduction has not been achieved. R.C. Baker recorded a technology where the demodulator reference signal (for coil current compensation) was used to eliminate orthogonal interference, which has been gradually replaced due to excessive power consumption [17]. In recent years, weak signal detection technology based on the correlation detection principle has developed rapidly. With its unique and simple signal processing method, lock-in amplification, with its unique and simple signal processing method, can extract the effective signal from an environment with strong noise and is widely applied in industry [18,19]. Although lock-in amplification technology can improve the flow measurement performance of an electromagnetic flowmeter under special working conditions, the induction electromotive signal of an electromagnetic flowmeter is not the ideal symmetry because of the different kinds of interference in the induction electromotive signal, and the processing method of single lock-in amplification still needs to be improved. In this paper, based on correlating previous research, electromagnetic flow measurement technology based on differential correlation detection is proposed. This can further extract effective flow signal from weak signal. The measurement performance of the electromagnetic flowmeter was improved under the conditions of low flow rate, strong external interference, and slurry interference. It provides a new research direction in the elimination of interference, reduction in measurement error, and improvement of measurement accuracy.

The following parts are organized as follows: part two proposes the electromagnetic flow measurement principle of the differential correlation detection, part three introduces the design of the platform construction and the experimental method, part four analyzes the experiment data, and part five contains the conclusion. 

## 2. Electromagnetic Flow Model Based on Differential Correlation Detection

### 2.1. Analysis of the Inter-Electrode Interference of the Electromagnetic Flowmeter

The inter-electrode interference of the EMF is very complex, and these interference signals are often mixed with the actual flow signal, leading to complex data-processing conditions. In order to improve the measurement accuracy of the EMF, the causes of these interference signals must be analyzed and summarized, and anti-interference measures must be taken to eliminate interference.

Currently, scholars from all over the world carry out studies on the inter-electrode signals of the EMF [20,21,22,23,24,25]. According to the different mechanisms, these signals can be classified into three categories.

The first one is the flow signal, differential interference, and in-phase interference based on the electromagnetic induction principle. The flow signal, as the effective signal needing to be measured, is used to show the actual flow rate in the pipeline. Differential interference signal is the sudden change in magnetic flux in a circuit composed of an electrode and its lead wire. It is also the induction electromotive force signal produced by Faraday’s law that blocks the change in the magnetic field; in-phase interference is the interference signal produced by the change in the secondary magnetic flux because the induction voltage produced by orthogonal interference can be coupled with the excitation coil.

The second category includes the polar interference, slurry interference, and flow interference which are related to electro-chemistry. Polar interference is closely related to all kinds of electro-chemical reactions, and is influenced by temperature and concentration etc. It is a gradually changing drift signal with a low frequency. Slurry interference is caused by the sudden change in voltage at the electrodes A and B when the solid particles move with the flowing fluids and cover the electrode interface or damage the oxide layer of the electrode surface. Due to different changes in polarization voltages at the two electrodes, the spike differential mode jump signal is superimposed on the output signal. The flow noise is caused by the difference in the number of charges at the electrodes A and B when measuring liquid with a low conductivity.

The third category is the power-line interference that is introduced into the system from an external circuit. It can be divided into serial mode interference and common mode interference. Serial interference is mainly caused by the dramatic magnetic flux leakage of the alternating magnetic field near the electromagnetic flow measurement system. Common mode interference is interference signal with the same polarity and amplitude in the two electrodes due to the difference between the actual ground and the ideal ground.

Currently, circuit compensation (hardware processing) and software filtering are mainly applied to eliminate interference in the field of EMF to improve measurement accuracy [26,27,28,29]. However, new interference is easily caused by circuit compensation. Moreover, various interferences are mixed in the original sampling signal, so that the actual flow signal cannot be obtained by software filtering, leading to the low accuracy or even failure of the EMF in the measurement. Periodic transmission and randomness in the signal are applied by the correlation detection principle to reduce the bandwidth and suppress noise to the largest extent, and finally the weak signal detection can be realized. The correlation detection principle is one of the most important methods to greatly improve the measurement accuracy of the traditional EMF. 

### 2.2. Differential Correlation Detection Principle

**Figure 1:**
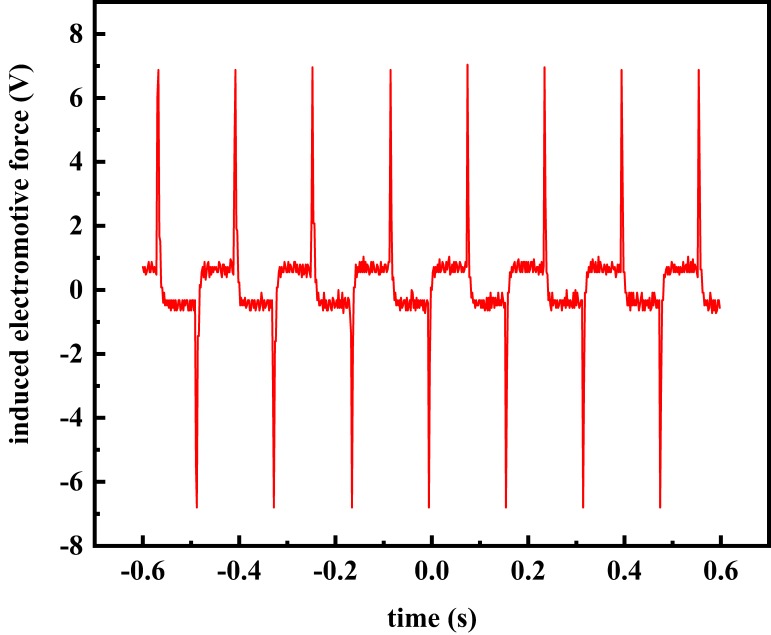
Induction electromotive signal.

Low frequency rectangular wave excitation is widely used in EMFs because it can reduce the influence of orthogonal interference on signal sampling [30,31]. Therefore, the low-frequency rectangular wave was selected as the excitation mode of the EMF based on the differential correlation principle. Then, the inter-electrode induction electromotive force was supposed to be a square signal with the same frequency as the excitation signal, as shown in Figure 1.

**Figure 2:**
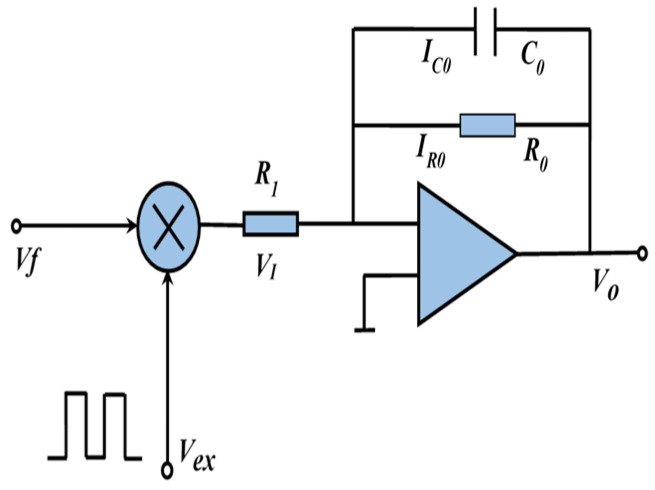
Correlation detection principle of the electromagnetic flowmeter (EMF).

The excitation square wave with a symmetric amplitude and the same frequency as the induction electromotive signal was used as the reference signal in order to make the noise elimination based on the correlation detection principle easier. The diagram of the EMF based on the correlation detection principle is shown in Figure 2. The multiplication of the induction electromotive signal *V_f_* and the excitation reference signal *V_ex_* is implemented in the switch amplifier. *V_I_* is the output of the multiplier and also the input of the integrator. *V_O_* is the output of the integrator. The excitation reference signal can be expressed in Equation (1):

(1) $$V_{ex}=\frac{4}{\pi }\sum_{n=0}^{\infty }\frac{1}{2n+1}\sin\left(2n+1\right)\cdot \omega_{f}t\;,$$

where *ω_f_* is the angular frequency of the excitation reference signal, n is a natural number, and *t* is the integration time. The electrode output signal includes the flow signal and interference signal. Since the interference signal and flow signal are not related, the electrode output signal of the electromagnetic flowmeter can be expressed in Equation (2):

(2) $$V_{f}=\frac{4}{\pi }V_{am}\sum_{n\to 0}^{\infty }\frac{1}{2n+1}\sin\left(2n+1\right)\cdot \omega_{R}\left(t+\tau \right)+n\left(t\right)\;,$$

where *ω_R_* is the angular frequency of the electrode output signal, *τ* is the delay time of the output signal related to the excitation reference signal, *V_am_* is the amplitude of the electrode output rectangular wave, and *n(t)* is the noise mixed in with the electrode output signal. The integrator input voltage *V_I_* and the output voltage *V_O_* satisfy the differential Equation (3):

(3) $$C_{0}\frac{dV_{O}}{dt}+\frac{V_{O}}{R_{0}}=-\frac{V_{I}}{R_{1}}\;,$$

By simplifying Equation (3), Equation (4) can be obtained.

(4) $$\frac{dV_{O}}{dt}+\frac{1}{R_{0}C_{0}}V_{O}=\frac{V_{I}}{C_{0}R_{1}}\;.$$

A first-order linear differential equation can be obtained by simplifying Equation (4), and the general solution can be obtained, as shown in Equation (5):

(5) $$V_{O}=\exp\left(-\int_{0}^{t}\frac{dt}{R_{0}C_{0}}\right)\left[\int_{0}^{t}\left(-\frac{V_{I}}{R_{1}C_{0}}\right)\cdot \exp\left(\int_{0}^{t}\frac{dt}{R_{0}C_{0}}\right)dt+C\right],$$

where *R_0_* and *R_1_* are the resistors of the integrator, *C* is the constant in the differential equation, and the initial voltage of the *C_0_* is zero. The output signal *V_I_* of the integrator can be obtained by multiplying *V_f_* and *Vex* in the multiplier, which can be expressed in Equation (6).

(6) $$\begin{array}{cc} V_{I} & =V_{f}\cdot V_{ex}\\ & =\left[\frac{4}{\pi }V_{am}\overset{\infty }{\underset{n=0}{\sum }}\frac{1}{2n+1}\sin\left(2n+1\right)\omega_{R}\left(t+\tau \right)+n\left(t\right)\right]\times \frac{4}{\pi }\overset{\infty }{\underset{n=0}{\sum }}\frac{1}{2n+1}\sin\left(2n+1\right)\omega_{R}t\\ & =\frac{4}{\pi }V_{am}\overset{\infty }{\underset{n=0}{\sum }}\frac{1}{2n+1}\sin\left(2n+1\right)\cdot \omega_{R}\left(t+\tau \right)\times \frac{4}{\pi }\overset{\infty }{\underset{n=0}{\sum }}\frac{1}{2n+1}\sin\left(2n+1\right)\omega_{R}t\\ & +n\left(t\right)\cdot \frac{4}{\pi }\overset{\infty }{\underset{n=0}{\sum }}\frac{1}{2n+1}\sin\left(2n+1\right)\cdot \omega_{R}t \end{array}$$

where the product of *n(t)* and the excitation reference signal is:

(7) $$n\left(t\right)\cdot \frac{4}{\pi }\sum_{n=0}^{\infty }\frac{1}{2n+1}\sin\left(2n+1\right)\cdot \omega_{R}t$$

In order to make the deduction easier, suppose *n(t) = V_N_sinω_N_t*, where *V_N_* is the amplitude of noise, *ω_n_* is the noise frequency (the superposition of the noise frequency and flow signal frequency in the electromagnetic flowmeter can be neglected). Equation (8) can be obtained:

(8) $$V_{N}\sin\omega_{N}t\cdot \frac{4}{\pi }\sum_{n=0}^{\infty }\frac{1}{2n+1}\sin\left(2n+1\right)\omega_{R}t\;.$$

According to the basic research of cross-correlation theory [32,33], when the frequency of the correlator and the frequency of the electromagnetic flowmeter is equal and the integration time is long enough, the correlation result of *n(t)* and the excitation signal is 0. Therefore, the Equation (6) can be simplified as Equation (9).

(9) $$\begin{array}{cc} & V_{I}=V_{f}\cdot V_{ex}\\ & =\frac{4}{\pi }V_{am}\sum_{n=0}^{\infty }\frac{1}{2n+1}\sin\left(2n+1\right)\cdot \omega_{R}\left(t+\tau \right)\times \frac{4}{\pi }\sum_{n=0}^{\infty }\frac{1}{2n+1}\sin\left(2n+1\right)\omega_{R}t \end{array}\;.$$

By substituting Equation (9) into Equation (5), Equation (10) can be obtained.

(10) $$V_{O}=-\frac{8R_{0}V_{\alpha m}}{\pi^{2}R_{1}}\left[1-\exp\left(-\frac{t}{R_{0}C_{0}}\right)\right]\sum_{0}^{\infty }\frac{1}{{\left(2n+1\right)}^{2}}\cos\left(2n+1\right)\omega_{R}\tau \;.$$

According to the infinite progression:

(11) $$y=\frac{C}{2}-\frac{4C}{\pi^{2}}\left[\cos\frac{\pi y}{C}+\frac{1}{3^{2}}\cos\frac{3\pi y}{C}+\frac{1}{5^{2}}\cos\frac{5\pi y}{C}+\ldots \right](0<y<C)\;,$$

where C is any constant. In order to make the calculation easier, substitute $y=\frac{C\omega_{R}\tau }{\pi }$ into Equation (11), and Equation (12) can be obtained.

(12) $$\frac{4}{\pi^{2}}\sum_{n=0}^{\infty }\frac{1}{{\left(2n+1\right)}^{2}}\cdot \cos\left(2n+1\right)\omega_{s}\tau =\left(\frac{1}{2}-\frac{\omega_{R}\tau }{\pi }\right)\;.$$

By substituting Equation (12) into Equation (10), Equation (13) can be obtained.

(13) $$V_{O}=-\frac{R_{0}V_{am}}{R_{1}}\left[1-\exp\left(-\frac{t}{R_{0}C_{0}}\right)\right]\left[1-\frac{2\omega_{R}\tau }{\pi }\right]\;.$$

where t is the testing time and $R_{0}C_{0}$ is the integration time, which can be expressed by Tc  φ. is the phase difference between the electrode output signal and excitation reference signal. When $t>>Tc=R_{0}C_{0}$, and $\varphi =\omega_{R}\tau$, Equation (14) can be obtained:

(14) $$V_{am}=-\frac{\pi R_{1}}{\left(\pi -2\varphi \right)R_{0}}\cdot V_{O}\;.$$

Therefore, the flow signal amplitude *V_am_* is related to the output DC voltage of the correlator *V_O_* and the reciprocal of the low pass filter gain *R*_1_/*R*_0_ as well as the phase difference between the electrode output signal and excitation signal φ. Due to the interference, the output signal cannot be absolutely symmetric. While improving the accuracy by applying the single lock-in amplification, new measurement errors are easily introduced into the system because of the phase difference. Therefore, the single lock-in amplification cannot obtain the actual flow accurately.

As for the single lock-in amplification, under ideal conditions—when the excitation reference signal and induction electromotive signal have the same frequency and phase—the output signal in the electrode is turned downward. When the two signals have the same frequency and the phase difference is 180 degrees, the output signal is turned upward. When the two have the same frequency and a phase difference of 90 degrees or 270 degrees, the average value is zero. In the practical applications, the induction electromotive signal is not absolutely symmetrical. When the phase difference is 90 degrees, the noise signal can be obtained. When the phase difference is 0, the effective signal mixed with noise can be obtained. Based on this theory, a method for differential correlation detection is proposed in this paper.

Two correlators are used in the system to realize the differential structure. A reference signal with the same phase as the flow signal is needed in correlator 1, and a reference signal which has a phase difference of 90 degrees to the flow signal is needed in correlator 2. Then, it can be seen that:

(15) $$V_{am1}=S\left(t\right)=s\left(t\right)+n\left(t\right)=\frac{\pi R_{0}}{\left(\pi -2\varphi_{0}\right)R_{1}}V_{O}\;,$$

(16) $$V_{am2}=n\left(t\right)=\frac{\pi R_{0}}{\left(\pi -2\varphi_{90}\right)R_{1}}V_{O}\;.$$

According to the differential amplification, Equation (17) can be obtained:

(17) $$\begin{array}{cc} s\left(t\right) & =V_{am1}-V_{am2}=S\left(t\right)-n\left(t\right)\\ & =\left(\frac{1}{\left(\pi -2\varphi_{0}\right)}-\frac{1}{\left(\pi -2\varphi_{90}\right)}\right)\frac{\pi R_{0}}{R_{1}}V_{O}=\left(\frac{2\left(\varphi_{0}-\varphi_{90}\right)}{\left(\pi -2\varphi_{0}\right)\left(\pi -2\varphi_{90}\right)}\right)\frac{\pi R_{0}}{R_{1}}V_{O} \end{array}$$

where *V_am1_* is the noise *S(t)*, including the noise *n*(*t*), and flow rate signal *s(t)*; *V_am2_* is the noise signal *n(t)* obtained by correlator 2. Finally, the actual flow signal *s(t)* can be obtained by subtracting *n(t)* from *S(t)*. $\varphi_{0}$ is the phase difference, which is equal to 0, and $\varphi_{90}$ is a phase difference of 90 degrees. After processing the signal with the differential method, the actual flow rate signal, which reduces the interference caused by signal asymmetry, can be effectively obtained.

**Figure 3:**
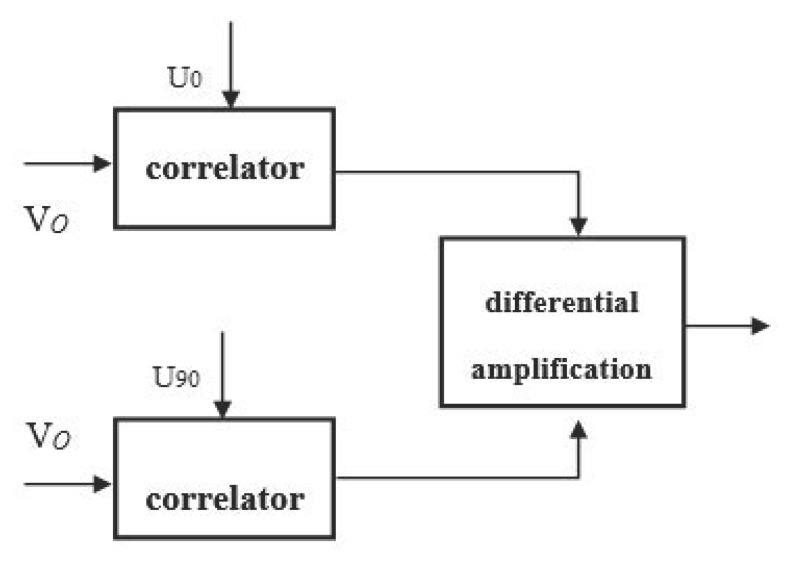
Structure of the differential correlator.

The structure of the differential correlator is shown in Figure 3. *V_0_* is the flow signal, *U_0_* is the excitation reference signal, and *U_90_* is the reference signal which has a 90 degrees phase difference to the flow signal. Since the rectangular wave is used for phase shifting, the resistance-capacitance (RC) active phase-shifting network cannot work well on phase shifting. If the phase-shifting circuit is composed of an integrated phase-locked loop circuit and D flip-flop, a rectangular wave of 90 degrees phase shifting can be obtained in theory. However, since the excitation frequency of the integrated phase-locked loop circuit itself needs to be considered and the appropriate resistance and capacitance values configured, unnecessary noise will be introduced, which is not conducive to the extraction of weak signals. In this system, the enhanced pulse width modulator (ePWM) module of digital signal process (DSP) is used to realize the 90 degrees phase shift of the square wave, which effectively solves the above problems.

These two correlator differential structures embody the core idea of anti-interference in this correlation detection flowmeter. No matter how the noise is generated and how the interference varies, the variation in the noises can always be tracked, and the noise can be eliminated after being processed by the differential correlation detection. This is more rapid and more effective than other noise feedback methods. The obtained flow signal is much more stable, especially at the zero-point stable part of the signal. This will be verified in the experiments introduced in the following parts.

## 3. System Design and Experiment Method

### 3.1. Electromagnetic Flowmeter Based on the Differential Correlation Principle

**Figure 4:**
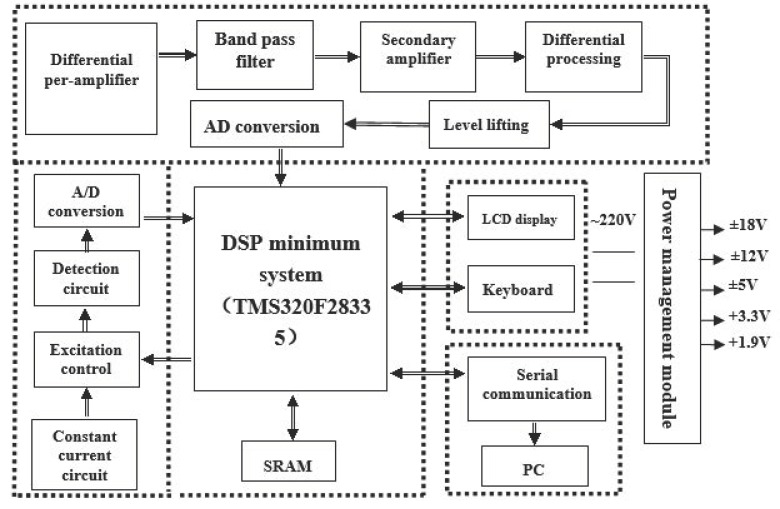
Diagram of the electromagnetic flowmeter based on differential correlation detection.

In order to ensure the accuracy of the experimental measurements, the framework of the electromagnetic flow measurement system based on the differential correlation detection principle was designed as shown in Figure 4.

The system hardware mainly included an excitation circuit of the electromagnetic flow sensor and an optimized detection and acquisition circuit of the electromagnetic flow signal. The digital signal processor TMS320F28335 (made by Texas Instruments, Dallas, United States of America) produced by Texas Instrument was the core of the excitation circuit and conversion. The hardware system module mainly included an excitation control module, a signal conditioning and acquisition module, a data processing and control module, a human–machine interface module, a communication module, and a power management module.

### 3.2. Experimental Platform

**Figure 5:**
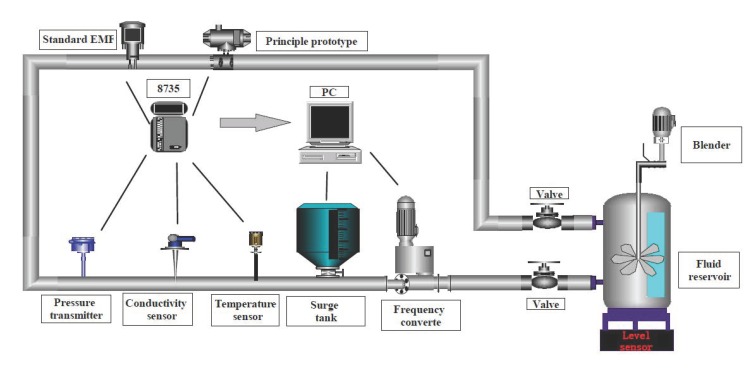
System diagram of the experimental platform.

The experiment was completed in the key laboratory of petroleum and natural gas equipment at the Ministry of Education (Southwest Petroleum University). The experimental platform was mainly composed of a liquid storage tank, a surge tank, a frequency converter, a pipeline centrifugal pump, a standard electromagnetic flowmeter, an electromagnetic flow measurement system based on differential correlation detection, various parameter sensors, and a data acquisition module. The experimental platform is shown in Figure 5.

The working process of the experimental platform was as follows: the pipe valve was opened, and the working frequency of the frequency converter was adjusted. The centrifugal pump rotated driven by the motor to extract the liquid in the liquid storage tank. After passing through the surge tank, a flow with a stable pressure could be formed. Then, the flow passed through an ADMAG AXW standard electromagnetic flowmeter (made by Yokogawa Electric Co., Ltd. Tokyo, Japan) with an accuracy level of 0.5 and a self-made electromagnetic flow measurement prototype based on the differential correlation detection principle. Taking the standard electromagnetic flowmeter as a reference, the performance of the self-made electromagnetic flowmeter was verified and compared. Then, the pipeline liquid returned to the liquid storage tank to form a closed circulation system. At the same time, the system parameters were transmitted to the computer through the data acquisition card PCI8735 (made by Chuanyi Automation Co., Ltd. Chongqing, China) to realize the monitoring of the whole cycle system.

### 3.3. Experiment Method

In order to verify the relationship between the output electromotive force and the flow rate of the differential correlation detection electromagnetic flowmeter, a flow calibration experiment with clean tap water was designed. By changing the working frequency of the frequency converter, the flow rate of the fluid in the pipeline could be adjusted. Fourteen data acquisition points were set up to record the value of the induced electromotive force output by the standard meter and the self-made flow measurement system based on the differential correlation detection principle.

In order to obtain the measurement accuracy of the instantaneous flow of the differential correlation detection electromagnetic flow measurement system, it was necessary to carry out verification experiments. By referring to the verification regulation of the JJG1033-2007 verification regulation on electromagnetic flow meters in China, the electromagnetic flow measurement system based on differential correlation detection was verified. Firstly, the whole system was preheated for 60 s. Then, we opened the pipeline valve, started the circulation system, adjusted the working frequency of the frequency converter, set six different flow test points, and tested the relevant parameters of the fluid (including conductivity, temperature, and pressure). After the output of the standard meter and the relevant electromagnetic flow measurement system were stable, the data was read and recorded. The following points were tested in the same way. Three tests were conducted for each test point, and the average value was taken and recorded. After all the tests were finished, the verification system was turned off.

In order to verify the superiority of the electromagnetic flow measurement technology based on differential correlation detection under the conditions of strong noise interference, slurry interference, and low flow rate, the self-made electromagnetic flow meter based on differential correlation, the standard electromagnetic flow meter, and the traditional electromagnetic flow meter were compared, and the average value was taken as the measurement value. The performance of the EMF based on the differential correlation principle was analyzed and tested.

## 4. Results and Analysis

The structure of this part is as follows. First, the calibration method and results of the electromagnetic flowmeter based on differential correlation are introduced; then the measurement accuracy of the instantaneous flow of the differential correlation detection electromagnetic flow measurement system is analyzed; finally, according to tests under the conditions of external strong interference, slurry fluid, and low flow rate, the actual data are given and analyzed.

### 4.1. Clean Water Calibration Experiment

#### 4.1.1. Calibration Method

The calibration method could be divided into real flow calibration and non-real flow calibration, according to the existence of the flows inside the pipeline [34,35]. Real flow calibration is widely used in small diameter pipeline flow measurements because of its simple principle and high accuracy. When the diameter of the pipeline increases, there are many problems in the real flow calibration, such as excessive energy consumption, the high cost of the standard device, and so on. Using non-real flow calibration is also a difficult and contentious topic in measurement problems of large-diameter electromagnetic flowmeters [36]. The pipeline used in this system had a diameter of 100 mm, which made it a small-diameter pipeline flow measurement. In order to lower the expense and implement measurements with a higher accuracy, real flow calibration was used in the system.

#### 4.1.2. Calibration Result

**Figure 6:**
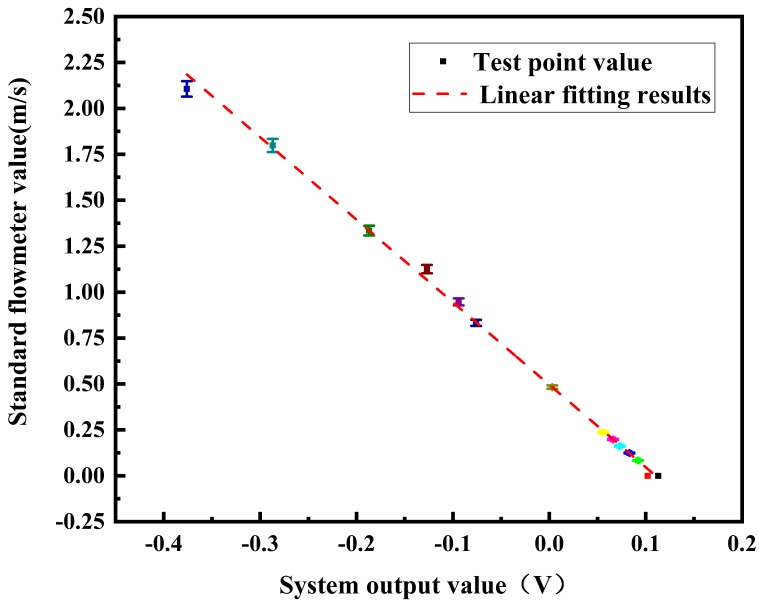
Relationship between the system output value and flow rate.

After the system was fully preheated, the working frequency was changed by the frequency converter to adjust the system flow rate. The variation in the flow rate changed the output effective voltage processed by the signal processing module. A data acquisition card was used to collect the output direct current voltage processed by the differential correlation detection technology, and the standard meter flow rate and the output electromotive force of electromagnetic flow measurement based on differential correlation detection were recorded at different test points. The relationship between the output voltage of the differential correlation detection electromagnetic flow system and the flow rate of the standard flowmeter is shown in Figure 6.

Through a data fitting analysis, the linear relationship between the output voltage of the differential correlation detection electromagnetic flow system and the flow rate is as shown in Equation (18).

(18) y=−4.49248x+0.49567

where *y* is the flow rate and *x* is the output voltage of the system. Linear regression coefficient *a* is −4.45405 and *b* is 0.49594, and the error range of *a* reaches −4.49248 ± 0.04729, while for *b* it is 0.49567 ± 0.00401, which are the meter factors of the electromagnetic flowmeter based on the differential correlation principle. It can be found that the output voltage of the tested flowmeter can linearly express the change in flow rate. The square value of the linear fitting index *R* is 0.99811, which meets the measurement requirements of electromagnetic flowmeters.

### 4.2. System Test Results

In order to obtain measurement accuracy for the instantaneous flow of the differential correlation detection electromagnetic flow measurement system, it was necessary to test the principle prototype. After the experimental platform was fully warmed up, the electromagnetic flow measurement system based on differential correlation detection was verified according to the regulations; the data obtained from the verification is shown in Table 1.

**Figure 7:**
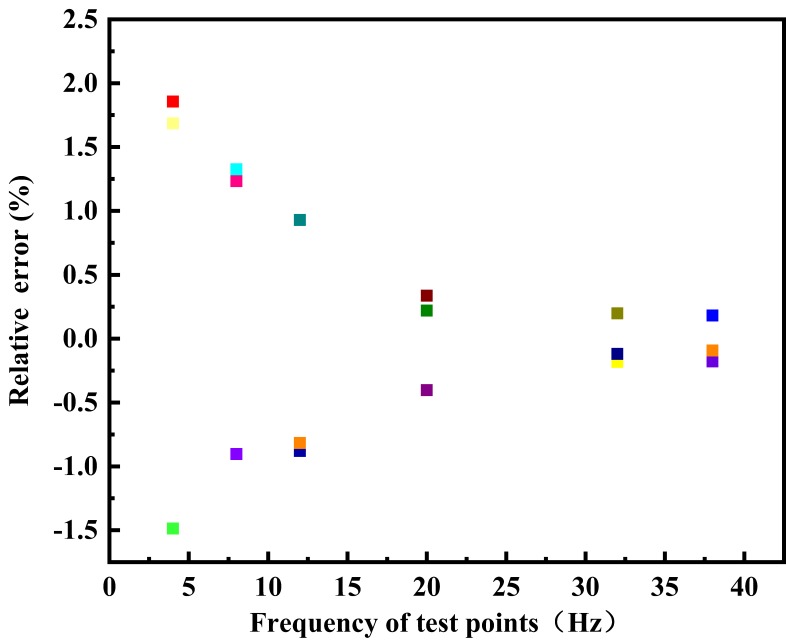
Distribution of the relative error of each test point in single verification.

**Figure 8:**
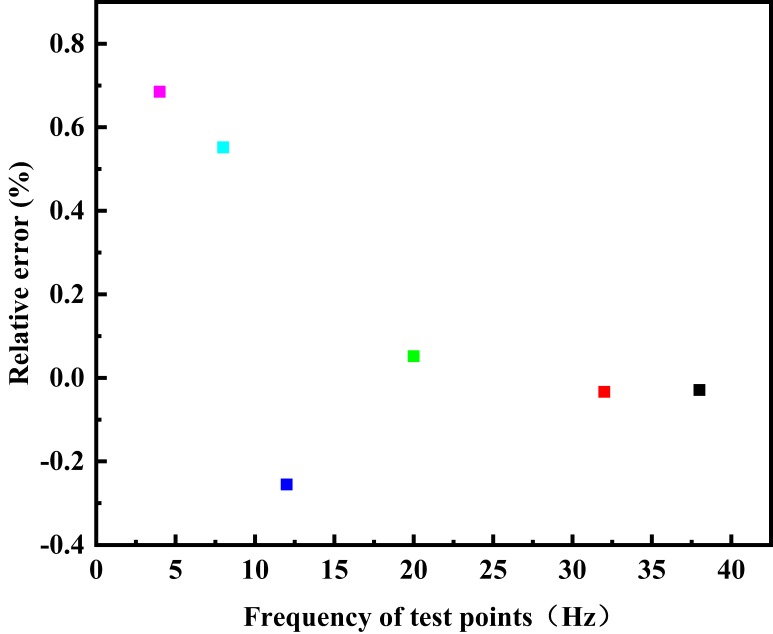
Distribution of the relative error of each flow test point.

**Figure 9:**
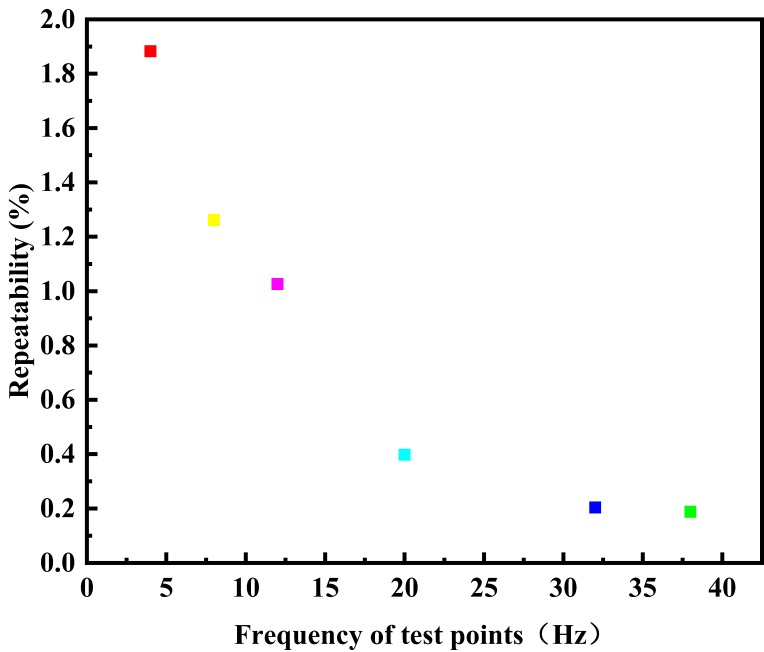
Distribution of the repeatability of each test point.

According to the test rules for the prototype of the electromagnetic flow measurement principle based on the differential correlation principle, the relative error distribution of each test point in a single test is shown in Figure 7, and the relative error distribution diagram of each flow test point is shown in Figure 8. If each test point is repeatedly measured under the same conditions, the repeatability distribution of the electromagnetic flow measurement system of differential correlation detection is shown in Figure 9.

It can be found from the curve distribution that when the flow rate is lower than 4.0 m^3^/h—that is, when the flow rate of the fluid in the pipeline is lower than 0.14 m/s—the relative error of a single test point is slightly higher than that of the conventional flow rate, and the relative indication error of a single point is mostly below 1.857%. When the flow rate is 4.6 m^3^/h–7.6 m^3^/h—that is, when the flow rate of fluid in the pipeline is 0.14m/s–0.25 m^3^/h—the maximum relative error of a single test point is only −1.326%, and the relative indication error of each test point is only 0.552%, which is higher than the measurement accuracy of the traditional electromagnetic flowmeter. The maximum relative error of the other single point is 0.929%, and the minimum relative indication error of the single point is −0.092%; the relative error of each flow test point fluctuates around 0%. When the flow velocity in the pipeline is lower than 0.25 m^3^/h, the repeatability is lower than that of the conventional flow velocity, and the flow repeatability of the other points fluctuates below 1.026%. When the flow is increased, the repeatability is gradually improved.

Based on the data analysis in Table 1, it can be seen that when the self-made electromagnetic flow measurement system based on differential correlation detection is within the flow measurement range of 4–54 m^3^/h, the maximum relative error of the system is 0.685%. This indicates that the instantaneous flow measurement accuracy level of the system is 1.0, which is a better performance than that of the traditional electromagnetic flowmeter.

### 4.3. Results and Analysis of Tests under Special Conditions

#### 4.3.1. Low Flow Rate Experiment

In order to test the response of the differential correlation detection electromagnetic flowmeter to a low flow rate, after the system was fully preheated, its ability to detect a low flow rate was quantitatively analyzed. When the frequency of the frequency converter was 1.8 Hz, the EMF based on the differential correlation principle had an apparent response. The frequency of the frequency converter was gradually increased from 1.8 Hz to 3.0 Hz in steps of 0.10 Hz, and the performance of the system was tested at a low flow rate of 0.084 m/s. The flow rate measured by the standard flowmeter and the frequency of the frequency converter were recorded. The output induction electromotive force was recorded by taking the average value of the data obtained in the experiments, as shown in Table 2.

**Figure 10:**
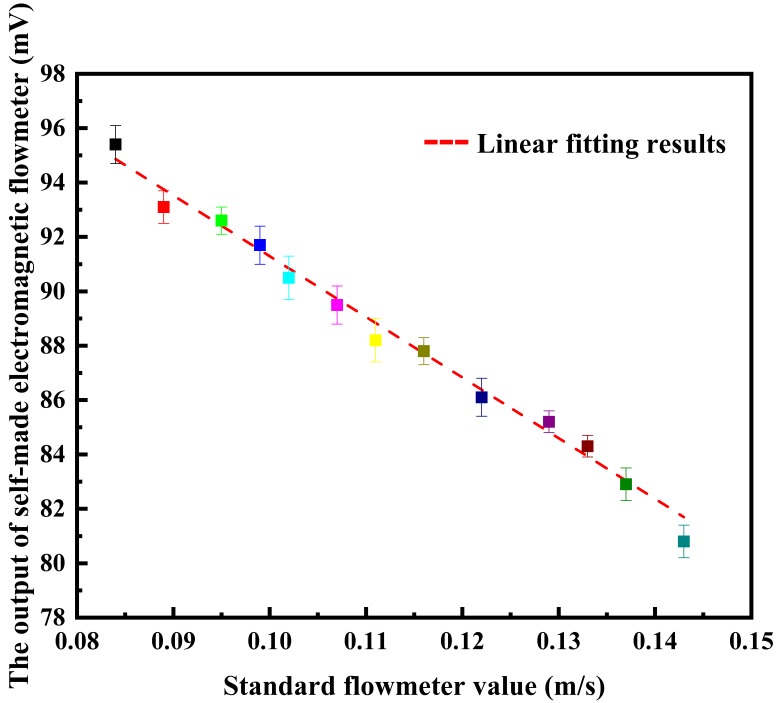
Relationship between the system output voltage and flow in the standard flowmeter.

According to the experimental data in Table 2, when the flow rate is lower than 0.143 m/s, the relationship between the output voltage of the electromagnetic flow measurement system based on differential correlation detection and the flow rate of the standard meter can be obtained as shown in Figure 10.

**Figure 11:**
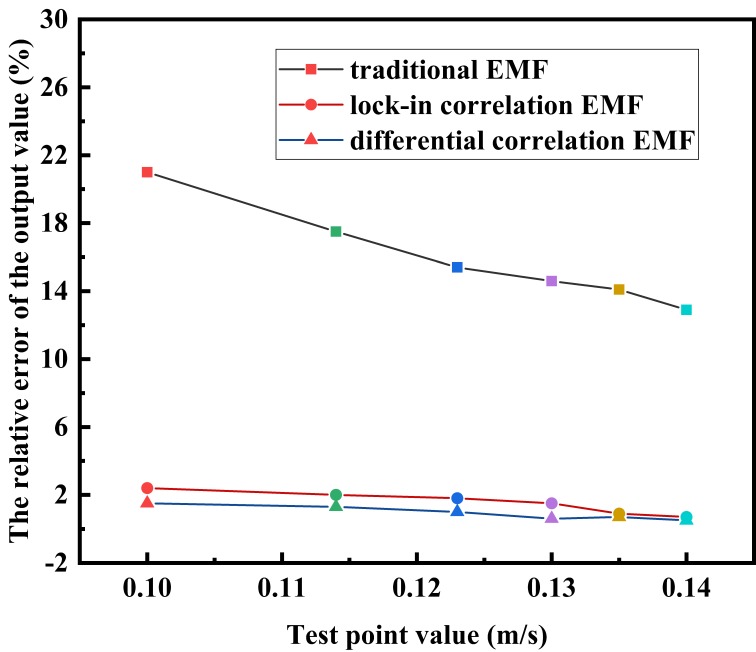
Relative error of the three flowmeters.

According to Figure 10, the fluctuation amplitude of the differential correlation detection electromagnetic flowmeter at each point in the low flow rate experiment can be obtained. Through the data fitting analysis, it can be seen that when the velocity is low, the signal output voltage of the differential correlation detection electromagnetic flowmeter has a good linear relationship with the flow rate, and the square value of the linear trend linear fitting degree index *R* is 0.98923. It can fully meet the requirements of the flow test at a low flow rate. In order to verify that the differential correlation detection technology can further improve the anti-interference ability of the electromagnetic flow detection system, the system was compared with the traditional electromagnetic flowmeter and the one based on the lock-in correlation system under the same experimental conditions. A high accuracy electromagnetic flowmeter was used to record the flow rate and served as the standard; the experimental data is shown in Table 3. According to the table, the relative error distribution of the three flowmeters was obtained, as shown in Figure 11.

From the error curve in Figure 11, it can be seen that the error of the traditional detection method not based on the correlation principle was significantly greater than that the other two methods. The error obtained from the detection method optimized by differential correlation detection was smaller and evenly distributed around zero. According to Table 3, the lower limit of the measured flow rate of the EMF based on the differential correlation principle can reach 0.084 m/s. When the frequency of the frequency converter increases from 1.8 Hz in steps of 0.1 Hz, the output has apparent variations. Therefore, the method has unique advantages in low flow rate measurement.

#### 4.3.2. Test under Strong External Interference

**Figure 12:**
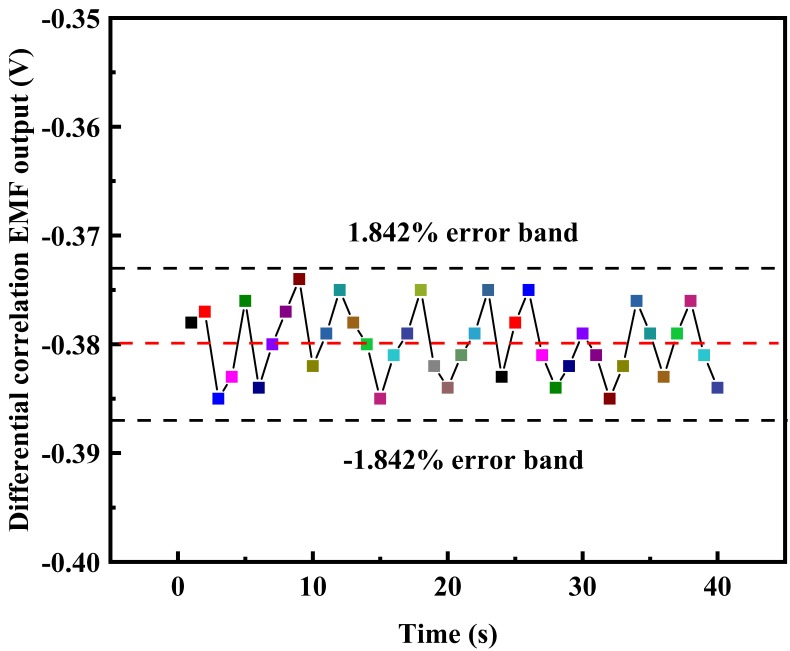
Test results of the EMF based on differential correlation detection under strong noise.

In order to verify the influence of strong external interference on the electromagnetic flowmeter based on the differential correlation principle, strong synchronous periodic pulse noise was introduced by turning on and off a high-power electric pump connected to the same power supply. Then, the random noise produced by the signal generator RIGOL DG 4062 (made by Chuanyi Automation Co., Ltd. Chongqing, China) was introduced into the system through the adder circuit to test the fluctuation rate of the system output under this condition. The test was conducted when the frequency of the frequency converter was set at 25 Hz. The test results are shown in Figure 12.

It can be concluded from Figure 12 that, due to the influence of strong external interference, the flow rate could not be stable, so the fluctuations could be very large. However, the performance of the tested instrument was consistent with that of the reference instrument. Therefore, in the evaluation of the developed differential correlation detection electromagnetic flowmeter, only the stable fluctuation rate of the amplitude was considered. From this point of view, in the test under strong external interference, the stable fluctuation rate of the amplitude of the tested instrument was less than 3.684%. The amplitude of the differential correlation detection electromagnetic flowmeter fluctuated around −0.379 V. According to previous research on the EMF based on the lock-in amplification correlation principle, it can be seen in Figure 12 that since the same noise in the signal could be eliminated by differential correlation detection, the signal to noise ratio of the signal was further improved, compared with the fluctuation of 15 mV in the experiment on lock-in amplification technology under strong external noise. The maximum fluctuation in the maximum value was only 10 mV, so the anti-interference ability and measurement accuracy were improved.

#### 4.3.3. Slurry Flow Test

**Figure 13:**
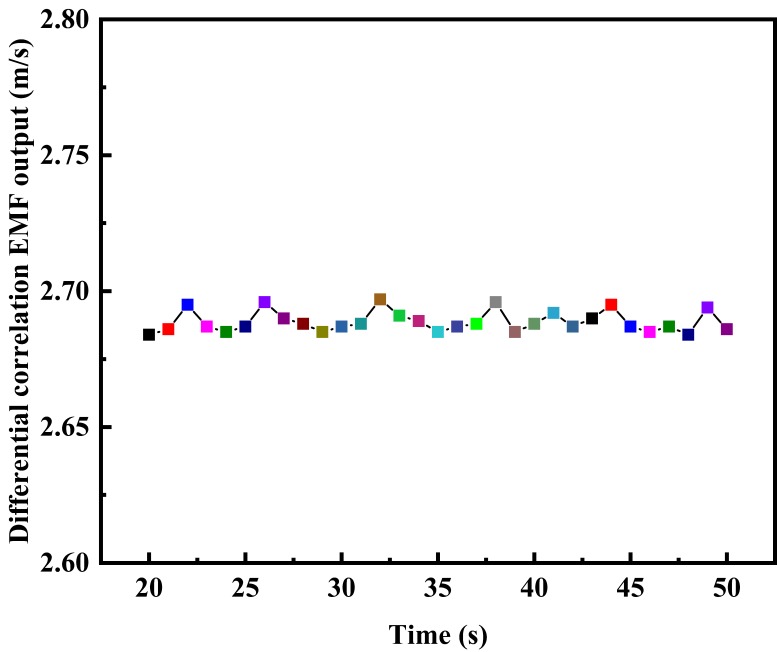
Test under slurry interference of the EMF based on differential correlation detection.

**Figure 14:**
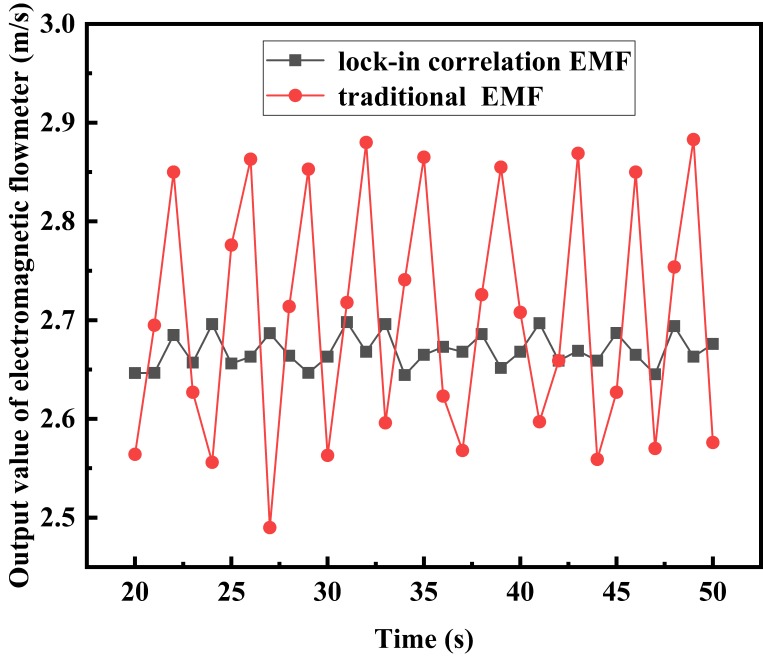
Test results of comparison experiments under slurry interference.

In order to test the influence of slurry interference on the differential correlation detection electromagnetic flowmeter, sand and bentonite (3 ‰) with a certain mass ratio were fully mixed in the tank and then added to the liquid storage tank. Then, the experimental platform was operated with full preheating to evenly distribute the slurry, the frequency of the frequency converter was set to 30 Hz, and the data was measured and recorded. The test results are shown in Figure 13. Under the same experimental conditions, comparison experiments for the traditional electromagnetic flowmeter and single lock-in correlation electromagnetic flowmeter were carried out for reference. The test results are shown in Figure 14.

It can be seen from Figure 13; Figure 14 that the traditional electromagnetic flowmeter is obviously affected by the slurry noise, and the flow rate fluctuates significantly between 2.491 m/s and 2.887 m/s. The flow rate of the single lock-in correlation electromagnetic flowmeter fluctuates significantly between 2.645 m/s and 2.6968 m/s. The electromagnetic flow measurement system based on differential correlation detection can effectively resist the impact of slurry interference, and stabilize the flow rate between 2.684 m/s and 2.696 m/s. The fluctuation amplitude is significantly smaller than that of the traditional electromagnetic flowmeter and lock-in correlation electromagnetic flowmeter. This method can suppress the slurry interference and improve the measurement accuracy.

## 5. Conclusions

This paper presents an electromagnetic flow measurement system based on the principle of differential correlation detection. Based on the analysis and tests above, the following conclusions can be drawn:

(1) In view of the demand for high accuracy electromagnetic flow measurement, a theoretical model of an electromagnetic flow measurement system based on differential correlation detection was proposed. The self-made electromagnetic flowmeter of the model system was researched and realized, and calibration and system test experiments were carried out. The results showed that the maximum relative error of the electromagnetic flow measurement system based on differential correlation detection was 0.685%, which could fully meet the requirements of the traditional electromagnetic flowmeter.
(2) In the slurry interference test, the electromagnetic flow measurement system of differential correlation detection could effectively resist the impact from slurry interference and stabilize the flow rate between 2.684 m/s and 2.696 m/s; the fluctuation amplitude was significantly smaller than that of the traditional electromagnetic flowmeter and electromagnetic flowmeter based on lock-in amplification.
(3) In a test under strong external interference, the signal-to-noise of the EMF based on differential correlation detection was further improved. The maximum fluctuation in the output electromotive force of the system was 10 mV, which enhanced the anti-interference ability of the system.
(4) In the low flow rate test, the error of the electromagnetic flow technology based on differential correlation detection was significantly smaller than that of the lock-in amplification technology and traditional electromagnetic flow meter. The flow rate lower limit of the electromagnetic flowmeter based on differential correlation detection could reach 0.084 m/s. When the frequency converter increased from 1.8 Hz to 3.0 Hz, the output of the method had obvious changes. This shows that the method has unique advantages in low flow rate measurement.

## Figures and Tables

**Table 1:** Data obtained from the flow test.

Flow Test Point (Frequency Hz)	Flow of Standard Meter (m^3^/h)	Flow of Tested Meter (m^3^/h)	Flow Difference (m^3^/h)	Relative Error of Each Point in Single test (%)	Relative Error of Each Verified Point (%)	Repeatability (%)
38	54.361	54.460	0.099	0.182	−0.029	0.188
	54.527	54.430	−0.097	−0.178		
	54.446	54.396	−0.050	−0.092		
32	43.641	43.562	−0.079	−0.181	−0.034	0.204
	43.702	43.789	0.087	0.199		
	43.689	43.637	−0.052	−0.119		
20	27.552	27.441	−0.111	−0.403	0.052	0.398
	27.563	27.656	0.093	0.337		
	27.631	27.692	0.061	0.221		
12	15.287	15.429	0.142	0.929	−0.255	1.026
	15.353	15.218	−0.135	−0.879		
	15.441	15.315	−0.126	−0.816		
8	7.637	7.568	−0.069	−0.903	0.552	1.261
	7.704	7.799	0.095	1.233		
	7.618	7.719	0.101	1.326		
4	4.573	4.505	−0.068	−1.487	0.685	1.883
	4.632	4.718	0.086	1.857		
	4.627	4.705	0.078	1.686		

**Table 2:** Low flow rate test of the differential structure.

Frequency (Hz)	Standard Flowmeter (m/s)			The Output of Self-Made Electromagnetic Flowmeter (mV)		
	min	max	avg	min	max	avg
1.8	0.083	0.085	0.084	93.9	96.1	95.4
1.9	0.088	0.090	0.089	92.5	94.2	93.1
2	0.094	0.096	0.095	91.8	93.1	92.6
2.1	0.098	0.101	0.099	90.7	92.4	91.7
2.2	0.101	0.103	0.102	89.6	91.3	90.5
2.3	0.106	0.108	0.107	88.5	90.2	89.5
2.4	0.100	0.102	0.111	87.4	89.7	88.2
2.5	0.114	0.117	0.116	86.6	88.3	87.8
2.6	0.119	0.124	0.122	85.4	87.6	86.1
2.7	0.128	0.131	0.129	84.8	86.1	85.2
2.8	0.132	0.135	0.133	83.2	84.7	84.3
2.9	0.136	0.138	0.137	82.1	83.5	82.9
3	0.141	0.145	0.143	80.4	81.6	81.0

**Table 3:** Relative error of the three flowmeters.

High Accuracy EMF Readings (m/s)	Relative Error of Traditional EMF (%)	Relative Error of Lock-in Correlation EMF (%)	Relative Error of Differential Correlation EMF (%)
0.100	21.0	2.4	1.5
0.114	17.5	2.0	1.3
0.123	15.4	1.8	1.0
0.130	14.6	1.5	0.6
0.135	14.1	0.9	0.7
0.140	12.9	0.7	0.5
